# *In Situ* Complexation of sgRNA and Cas12a Improves the Performance of a One-Pot RPA–CRISPR-Cas12 Assay

**DOI:** 10.1021/acs.analchem.4c01777

**Published:** 2024-06-12

**Authors:** Jake M. Lesinski, Thomas Moragues, Prerit Mathur, Yang Shen, Carolina Paganini, Léonard Bezinge, Bo Verberckmoes, Bodine Van Eenooghe, Stavros Stavrakis, Andrew J. deMello, Daniel A. Richards

**Affiliations:** † Institute for Chemical and Bioengineering, ETH Zurich, Vladimir-Prelog-Weg 1, 8093 Zürich, Switzerland; ‡ Institute of Food, Nutrition and Health, ETH Zurich, Schmelzbergstrasse 7, 8092 Zürich, Switzerland; § Faculty of Medicine and Health Sciences, Department of Public Health and Primary Care, Ghent University, De Pintelaan 185, 9000 Gent, Belgium

## Abstract

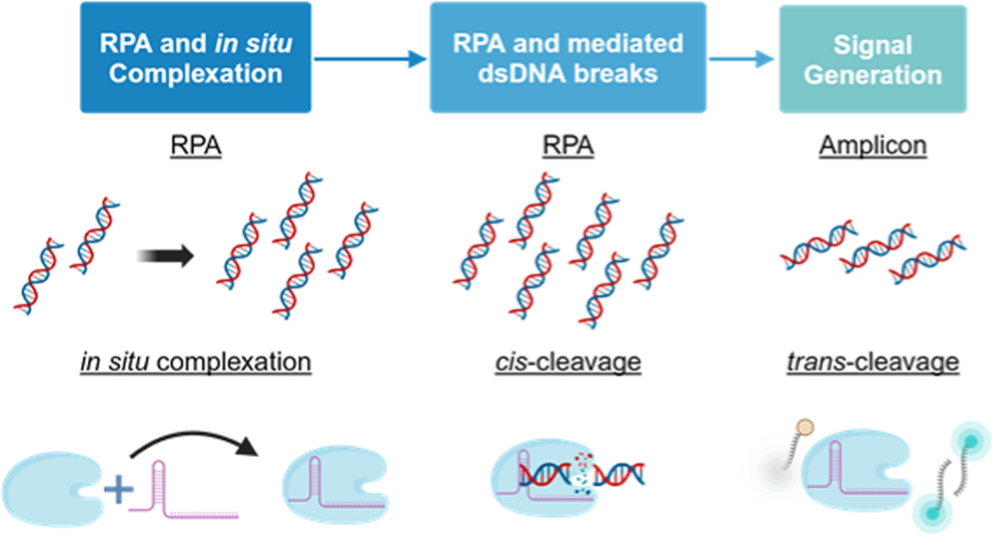

Due to their ability to selectively target pathogen-specific nucleic acids, CRISPR-Cas systems are increasingly being employed as diagnostic tools. “One-pot” assays that combine nucleic acid amplification and CRISPR-Cas systems (NAAT–CRISPR-Cas) in a single step have emerged as one of the most popular CRISPR-Cas biosensing formats. However, operational simplicity comes at a cost, with one-pot assays typically being less sensitive than corresponding two-step NAAT–CRISPR-Cas assays and often failing to detect targets at low concentrations. It is thought that these performance reductions result from the competition between the two enzymatic processes driving the assay, namely, Cas-mediated *cis*-cleavage and polymerase-mediated amplification of the target DNA. Herein, we describe a novel one-pot RPA–Cas12a assay that circumvents this issue by leveraging *in situ* complexation of the target-specific sgRNA and Cas12a to purposefully limit the concentration of active Cas12a during the early stages of the assay. Using a clinically relevant assay against a DNA target for HPV-16, we show how this *in situ* format reduces competition between target cleavage and amplification and engenders significant improvements in detection limit when compared to the traditional one-pot assay format, even in patient-derived samples. Finally, to gain further insight into the assay, we use experimental data to formulate a mechanistic model describing the competition between the Cas enzyme and nucleic acid amplification. These findings suggest that purposefully limiting *cis*-cleavage rates of Cas proteins is a viable strategy for improving the performance of one-pot NAAT-CRISPR-Cas assays.

## Introduction

Clustered regularly interspaced short palindromic repeats—CRISPR associated proteins (CRISPR-Cas) systems are promising tools for detecting specific nucleic acid sequences, particularly when combined with nucleic acid amplification techniques (NAATs) such as recombinase polymerase amplification (RPA).^1−3^ Although optimum assay sensitivity in NAAT–CRISPR-Cas assays is typically achieved when the two techniques are performed sequentially (*i.e.*, NAAT followed by CRISPR-Cas detection), “one-pot” formats have been developed to improve practicality, often at the expense of assay sensitivity.^4^ Previous reports have suggested that sensitivity reductions in one-pot systems are a result of the exonuclease activity (*cis*-cleavage) of the Cas-based ribonucleoprotein (RNP), which hydrolyses target DNA and hinders efficient nucleic acid amplification.^5^ Based on this hypothesis, we decided to explore whether deliberate modulation of the functional concentration of the Cas-based RNP at the start of the assay could be leveraged to control exonuclease activity and ultimately lead to improved signal generation in one-pot RPA–CRISPR-Cas12a assays. We evaluate the individual aspects of this approach, including the kinetics of RNP formation, *cis* exonuclease activity, and the diffusion of key reaction components to develop a mathematical model for determining optimal *cis* cleavage rates in RPA–CRISPR-Cas12 assays.

Since their genesis, a plethora of CRISPR-Cas-based diagnostic assays have been reported.^2,3^ Such assays leverage the ability of CRISPR RNAs (single-guide RNAs or sgRNAs) to guide Cas proteins to bind to, and cleave, specific nucleic acid targets (*cis*-cleavage). This “specific” cleavage is then followed by activation of the “non-specific” (*trans*-cleavage) pathway of the Cas protein and collateral cleavage of single-stranded DNA in the locality. Collateral cleavage can be exploited to generate a detectable signal, for example, by cleaving single-stranded DNA-quenched fluorophore reporters.^2,3^ For interested readers, an excellent overview of CRISPR-Cas diagnostics is provided by Shen et al.^6^ The explosion of interest in CRISPR-Cas diagnostics was catalyzed by the development of two seminal assays: the Specific High-sensitivity Enzymatic Reporter Unlocking (SHERLOCK)^2^ and DNA Endonuclease-Targeted CRISPR Trans Reporter (DETECTR)^3^ assays. Both SHERLOCK and DETECTR combine the exquisite target specificity of CRISPR technology with the rapid amplification capabilities of RPA.^3,4,7^ However, both share a common weakness, in that they require preamplification of the nucleic acid (with DETECTR also requiring preformation of the sgRNA–Cas ribonucleoprotein) to work efficiently. This makes both assays complex, multistep processes, poorly suited for automation, and prone to contamination. This limits their practicality, particularly when considering diagnostic applications within resource-limited settings.^8,9^

Realizing these limitations, several groups have sought to develop “one-pot” assays that reconcile NAAT and CRISPR-Cas processes within a single reaction vessel (Table S1). Key to these methods is mitigating the competition between nucleic acid amplification and exonuclease activity.^10−14^ An essential aspect of the CRISPR-Cas target recognition pathway is the irreversible cleavage of the target nucleic acid by the Cas ribonucleoprotein. This same target nucleic acid also serves as a template for nucleic acid amplification. Previous studies have suggested that at low nucleic acid concentrations, target cleavage can outcompete target amplification, thus hamstringing the entire signal generation cascade.^5,11,12^ The simplest methods for minimizing this competition involve initially separating the CRISPR and NAAT reactions within the same vessel and then combining them after a defined period. A popular way to achieve this is to store the CRISPR-Cas reaction components in the lid of a reaction tube and then perform an NAAT reaction in the bottom of the same tube. At a predetermined time, the reagents are mixed by centrifugation.^13,14^ A more elaborate method was developed by Hu et al., who presented a photocaged single guide RNA (sgRNA) that can only perform *cis*-cleavage after irradiation with ultraviolet light.^10,12^ Additionally, Lin et al. devised a one-pot assay in which CRISPR-Cas components are stored in a glycerol-containing buffer to purposefully slow their diffusion upon addition to the NAAT reaction, thus limiting *cis*-cleavage of the target.^11^ While these methods can mitigate some of the losses associated with one-pot NAAT–CRISPR-Cas assays, they introduce additional complexities and practical limitations. For example, methods relying on physical separation within the same tube are prone to premature mixing upon accidental agitation of the tubes, and photochemical methods require the synthesis of bespoke sgRNAs. Furthermore, these methods require additional human interventions, which is counterproductive to assay simplification and automation.

**Figure 1:**
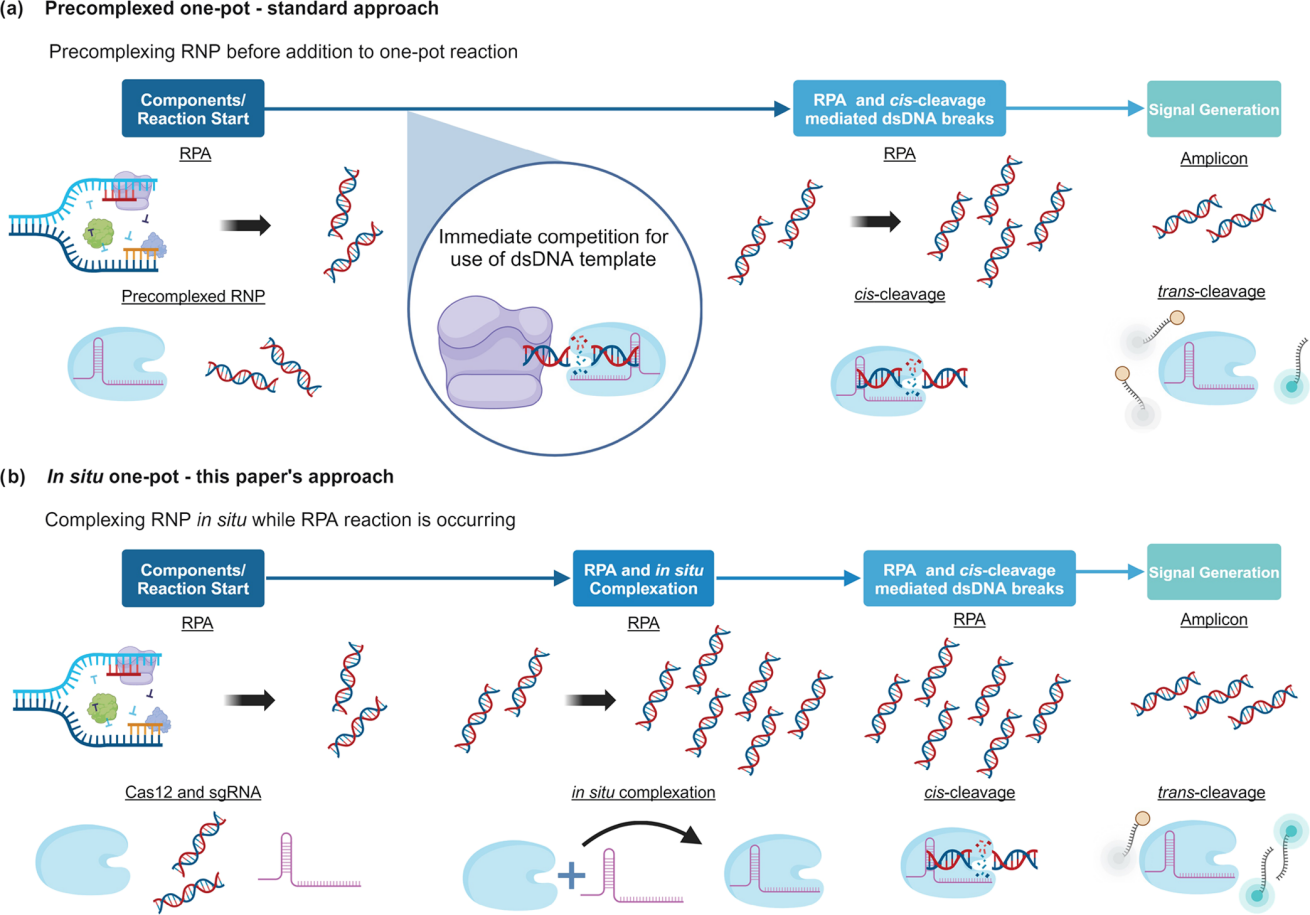
Diagrammatic representations of the precomplexed and *in situ* RPA–Cas12a one-pot formats. (a) The traditional one-pot format employing a precomplexed Cas12a-RNP that immediately competes with RPA for the target DNA. (b) The *in situ* one-pot approach presented in this paper, in which the formation of the RNP occurs over the course of the assay.

Herein, we introduce and investigate a straightforward method for increasing the sensitivity of one-pot RPA–CRISPR-Cas12 assays while simultaneously simplifying the assay workflow. Based on the competition theory outlined above, we hypothesized that by limiting the amount of active RNP complex present in the reaction mixture during the early stages of the reaction, we could shift competition in favor of nucleic acid amplification during this critical time frame (Figure 1). Furthermore, we theorized that if we could engineer a situation in which the concentration of RNP increased as the assay progressed, then the signaling benefits of high RNP concentrations would be maintained but without the drawbacks associated with early reaction *cis* exonuclease activity. We show that this can be achieved by purposefully omitting the oft-included preformation of the ribonucleoprotein and instead exploiting the slow association kinetics between Cas12a and the target-specific sgRNA inside the viscous RPA reaction medium. Significantly, our method requires no additional chemical additives, engineered proteins, or physical apparatus.

## Experimental Section

### Precomplexed *vsIn Situ* Complexed One-Pot Assays

Complexes were preassembled by mixing the following reagents at final concentrations of 250 nM Cas12a, 250 nM sgRNA (Microsynth AG, Balgach, Switzerland), 1× HOLMES buffer, and nuclease-free water (Thermo Fischer Scientific, Waltham, USA). These reagents were held at 37 °C for 30 min. The precomplexed one-pot reaction was prepared by mixing the following reagents at final concentrations of 25 nM Cas12a, 25 nM sgRNA (as an RNP), 0.1× HOLMES, 2 μM fluorescent reporter (*trans*-cleavage—Microsynth AG, Balgach, Switzerland), HPV16 RPA Primer Forward 480 nM (Microsynth AG, Balgach, Switzerland), HPV16 RPA Primer Reverse 480 nM (Microsynth AG, Balgach, Switzerland), 1× Twist Amp Basic Rehydration buffer and lyophilized pellet (TwistDx, Maidenhead, U.K.), 20 mM potassium acetate, nuclease-free water, and the target concentration of interest. The *in situ* one-pot mixture was created in the same manner as the precomplexed one-pot mixture; however, the sgRNA and Cas12a were added directly to the one-pot rather than as an RNP. Each sample (20 μL) was loaded into a black 384-well microtiter plate (Corning, Corning, USA) and covered with light mineral oil (5 μL) (Sigma-Aldrich, Burlington, USA), before being placed into a BioTek Synergy H1 Multimode Reader (Agilent, Santa Clara, USA). The reaction was allowed to proceed for 180 min at 37 °C, with fluorescence intensity (Ex_495±15_/Em_528±15_) being measured every 2 min.

### Analysis of Patient Clinical Samples

Clinical samples were collected by a gynecologist using a Viba brush (Rovers Medical Devices, Oss, Netherlands). The cervix and the superficial vaginal canal were swabbed with the brush, which then was rinsed in Hologic ThinPrep medium (Hologic, Mississauga, Canada). Cervical swabs were kept in Hologic ThinPrep medium, stored at 4–8 °C, and then concentrated and reconstituted in 200 μL of PBS with 1% (v/v) IGEPAL CA-630 (Sigma-Aldrich, Burlington, USA). Samples were analyzed using the precomplexed and one-pot procedures outlined previously.

### Fluorescence Correlation Spectroscopy (FCS)

FCS experiments were performed using a custom-built system. A continuous Genesis MX488–1000 STM laser operating at 488 ± 3 nm (Coherent, Saxonburg, USA) was adjusted to a power of 4 mW using an ND filter. The laser beam was coupled to a C2si confocal laser scanner mounted on an Eclipse Ti microscope (Nikon, Egg, Switzerland), equipped with a 20 μm pinhole. The sample was placed in a 384-well plate with a coverslip bottom (Azenta Life Sciences, Berlin, Germany) and measured through a 60X/1.2 NA water immersion objective (Nikon, Egg, Switzerland). Emitted photons were directed through an optical fiber, collimated with an F950FC-A laser collimator (Thorlabs, Bergkirchen, Germany), passed through a 525/39 BrightLine emission filter (AHF, Tübingen, Germany), and focused onto a SPCM-AQRH single-photon counting detector (Excelitas Technologies, Waltham, USA) using an AC254–050-A doublet lens (Thorlabs, Bergkirchen, Germany). The microscope was controlled with NIS Elements C software (Nikon, Egg, Switzerland), and the data were collected with Symphotime64 software (PicoQuant, Berlin, Germany). The microscope and photon counting module were controlled by separate computers connected *via* a home network. Data were analyzed using a custom MATLAB (MathWorks, Natick, USA) code.

### Surface Plasmon Resonance (SPR)

Sensorgrams reporting the binding of the Cas proteins to immobilized sgRNA were obtained using a Biacore X surface plasmon resonance instrument (GE Healthcare, Glattbrugg, Switzerland). First, the streptavidin-coated surface of a SAD50L chip (Xantec Bioanalytics, Düsseldorf, Germany) was coated with 2 μM of sgRNA in the experimental flow cell at a flow rate of 5 μL/min. For kinetics studies, Cas12a protein samples of variable concentration were passed through both cells in an SPR running buffer at 10 μL/min and 25 °C. For each concentration, the association was measured over a period of 180 s, and the dissociation was monitored for 360 s. The surface was then regenerated using a 5 s injection of regeneration buffer (10 mM glycine, pH 2) at a flow rate of 10 μL/min. This cycle was repeated for each measurement. Data were fit to a 1:1 kinetic binding model, and the values for *k*_on_ and *k*_off_ were determined directly from the fits. The equilibrium dissociation constant (*K*_D_) was determined by dividing *k*_off_ by *k*_on_.

### *cis*-Cleavage Kinetics Investigation

The *cis*-cleavage reporter (Microsynth AG, Balgach, Switzerland) was formed by mixing *cis*-cleavage reporter–quench and *cis*-cleavage reporter–fluorophore to final concentrations of 1.5 μM in nuclease-free water. The thermal binding protocol started at 95 °C and cooled at a rate of 10 °C/min until a temperature of 4 °C was reached. Various final concentrations of this bound *cis*-cleavage reporter (80, 40, and 20 nM) were then mixed with Cas12a (to a final concentration of 2 μM), sgRNA (to a final concentration of 2 μM) in an RPA reaction buffer (to a final concentration of 1×), and nuclease-free water. Each sample (20 μL) was loaded onto a 384-well microtiter plate and covered with mineral oil (5 μL), before being placed into the plate reader. The reaction was allowed to run for 180 min at 37 °C, with fluorescence (Ex_495±15_/ Em_528±15_) being recorded every 2 min.

### Modeling of One-Pot Reactions

**Scheme 1:**
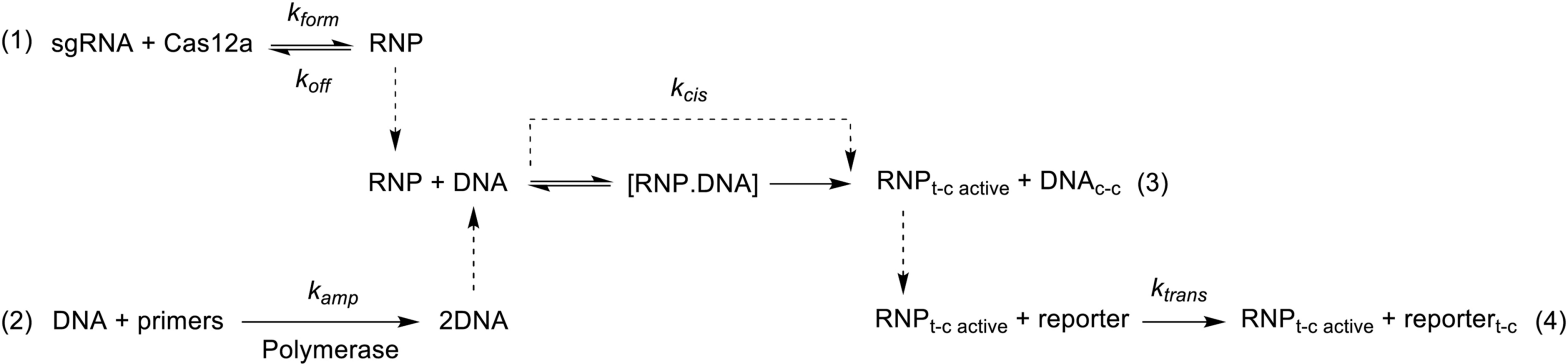
Overview of the Modelled Reaction Network Reactions 1–4 and 2–4 are considered in the *in situ* and precomplexed scenarios, respectively. *t*-c = *trans*-cleavage, *c*-c = *cis*-cleavage.

One-pot reactions were modeled in COMSOL Multiphysics version 6.0. Kinetics were computed using mass action laws over the duration of an experiment. The equations shown in Scheme 1 govern the precomplexed assay. Initial concentrations of RNP (25 nM), DNA (varied), enzyme (polymerase—unknown but assumed to be in great excess, 100 μM, and thus not expected to impact the amplification’s kinetics), primer (480 nM), and reporter (2000 nM) were fixed at the experimental values used in this work. The equations governing the *in situ* complexed model were the same as for the precomplexed scenario but with the addition of a step controlling the production of RNP (Scheme 1). *trans*-Cleavage turnover was set to 1.95 s^–1^, while the *cis*-cleavage rate was varied between *k* = 0.0024 and 0.054 s^–1^.^15,16^ Further, amplification (Scheme 1, **eq (2)**) was assumed to be a simple doubling rate having values between 100 and 18,000 M^–1^·s^–1^. Rates that illustrated reasonable amplification times alone (Figure S1) were selected. Both FCS data and diffusion modeling indicated that the reaction was not diffusion-controlled (Figure S2); therefore, diffusion was neglected. Due to the incompatibility of the SPR instrument with high-viscosity fluids, SPR was performed in a buffer that did not contain poly(ethylene glycol) (PEG) and several proteins found in RPA. With this in mind, we hypothesized that the actual *k*_on_ would be lower than that found from SPR. Thus, we used three values, starting with our experimental value of 175,000 M^–1^·s^–1^, lowering it to 17,500 and ultimately 1750 M^–1^·s^–1^.

## Results and Discussion

### Comparing Precomplexed and *In Situ* One-Pot Assays

**Figure 2:**
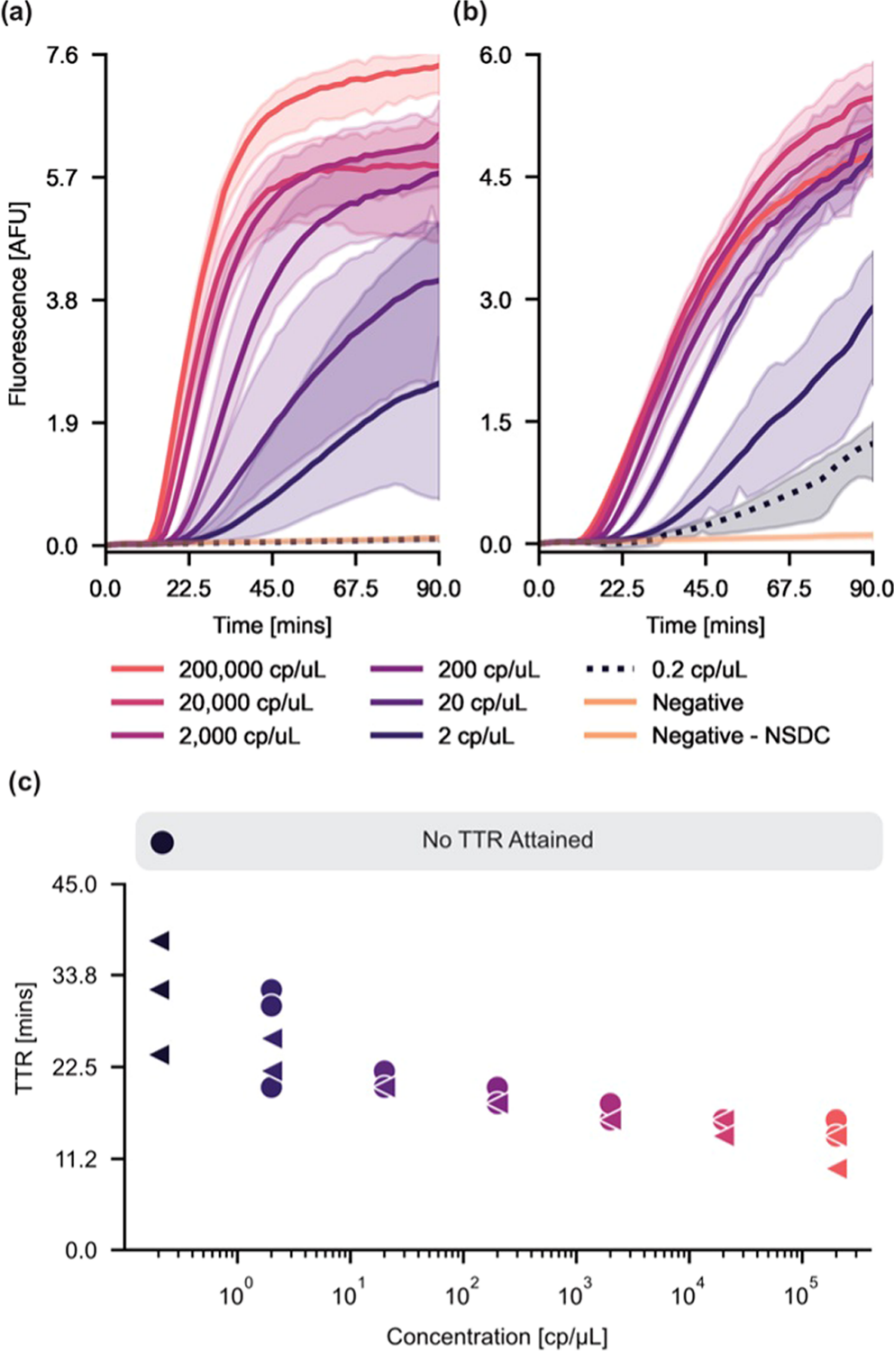
Performance comparison between precomplexed and *in situ* RPA–Cas12a assays. (a) A graph of fluorescence *vs* time for the precomplexed one-pot RPA–Cas12a assay. The assay was unable to detect the target at 0.2 copies/μL titer. (b) A graph of fluorescence *vs* time for the *in situ* one-pot RPA–Cas12a. All sample titers were detected. The negative curves correspond to a target concentration of 0 copies/μL, and the negative–NSDC represents a control with the addition of background DNA. (c) A graph of the time-to-result (TTR) *vs* concentration for the precomplexed (green circles) and *in situ* (blue triangles) assays. Time-to-result was determined according to an established slope-based algorithm.^20^ For the precomplexed RPA–Cas12a assay, no TTR was determined at a target concentration of 0.2 copies/μL. We found no significant differences in TTR between the two assays at all other target concentrations.

To begin, we decided to assess how omitting the precomplexation of the RNP impacts signal generation in one-pot RPA–CRISPR-Cas12a assays. To this end, we devised two model one-pot RPA–CRISPR-Cas12a assays. In the first, we preformed the RNP prior to mixing with the RPA reaction components (henceforth referred to as “precomplexed”), as described by Chen et al.,^3^ and in the second, we mixed all reaction components at the same time (henceforth referred to as “*in situ*”). As a target, we chose the L1-encoding gene of the *Human papillomavirus* (HPV-16) due to its extensive history as a model target for CRISPR-Cas-based assays^3,15,17,18^ and its clinical utility as a biomarker for cervical cancer.^19^ We monitored the *trans*-cleavage of a fluorescence reporter as a function of target concentration and time for each assay (Figure 2a,b), employing a slope-based algorithm to determine the time-to-result for each target concentration (Figure 2c).^20^ This algorithm takes the first derivative of the raw fluorescence signal for each sample and calls a sample as being “positive” once three consecutive readings differ from the first derivative of the negative at a distance of three standard deviations.

Using this algorithm, we found that the *in situ* one-pot assay was able to detect target concentrations down to 0.2 copies per microliter, whereas the traditional one-pot assay was only capable of detecting target concentrations at or above 2 copies per microliter. While the *in situ* one-pot assay was able to detect lower titers of DNA than the traditional one-pot assay employing a precomplexed RNP, the absolute signal was consistently lower. This is likely a result of the overall decreased RNP concentration, which would lead to a decrease in collateral *trans*-cleavage of the quenched fluorescent reporter. However, these data support our hypothesis that at lower DNA concentrations, the *cis* exonuclease activity of the Cas protein hinders efficient DNA amplification by RPA.

### Precomplexed One-Pot *vs In Situ* Complexed One-Pot–Clinical Application

The robustness of the *in situ* complexed assay was then investigated using patient-derived samples. Eight positive and eight negative HPV16 vaginal swabs were lysed and stored in universal transport media, before being analyzed using both our *in situ* complexed one-pot assay and the precomplexed one-pot assay. To obtain a reference standard, the samples were also analyzed using the Allplex HPV28 qPCR test. The data presented in Table 1 indicate that the *in situ* assay results agree more closely with the Allplex qPCR data than the precomplexed assay. In several samples (*e.g.*, 2, 3, 5, and 7), one or more false negatives were wrongly indicated by the precomplexed assay format; however, these were correctly indicated by the *in situ* complexed assay format. In one instance, seen in one of the three technical replicates of positive 8, the precomplexed format agreed with the Allplex result while the *in situ* complexed format did not. For both assays, all negative samples were correctly identified, that is, zero false positives. The high number of false negatives was likely caused by the lower sensitivity of our assays compared to PCR.

**Table 1:** Analysis of Samples Obtained from Patient Vaginal Swabs Using the Precomplexed and *In Situ* One-Pot RPA–Cas12 Assays Targeting the L1-Encoding Gene of HPV-16 in Technical Triplicates^b^

	time-to-result^a^ (TTR) (min)						
sample number	precomplexed			*in situ*^c^			Allplex (Ct)
1	22	22	20	26	24	24	21.63
2	36	60	ND^d^	20	18	18	32.70
3	ND	ND	ND	24	ND	52	29.38
4	ND	ND	ND	ND	ND	ND	33.66
5	ND	ND	ND	52	ND	40	30.80
6	24	24	ND	26	26	36	28.25
7	ND	22	22	24	24	22	21.26
8	ND	ND	36	ND	ND	ND	34.39
9–16^e^	NEG	NEG	NEG	NEG	NEG	NEG	NEG

a Time-to-result was determined using a slope-based algorithm, as described above.

b Precomplexed refers to the assay in which a fully preformed RNP was added to the assay mixture.

c *In situ* refers to the assay in which the RNP is formed *in situ*.

d ND = not detected.

e Samples 9–16 were taken from individuals confirmed as HPV-16 negative.

Interestingly, in the case of patient-derived samples, we observed no benefits in terms of TTR when employing the *in situ* protocol. No statistically significant differences were found between the *in situ* and precomplexed assays. We attribute this to the relatively small sample size and the large variation in TTR between technical replicates. This variation is likely due to the viscosity and inhomogeneity of the samples. Another explanation is that the titers of the HPV-16 target in these samples were too high to differentiate using TTR. Even in our idealized buffer system (Figure 2), we only observed differences in the TTR in the low-titer samples.

### Investigation of the Mechanism

**Figure 3:**
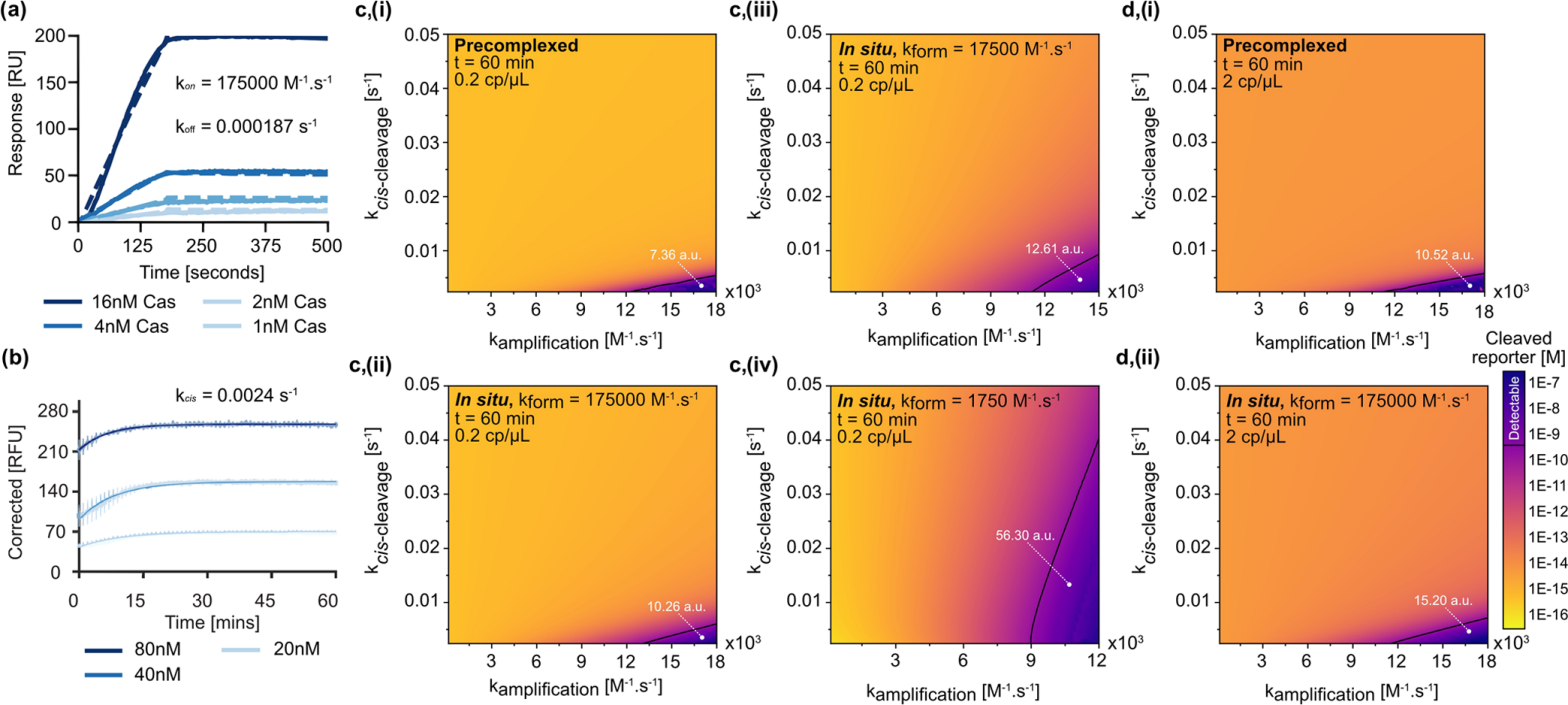
Experimental investigation and modeling of the competition between RPA and Cas12a-mediated *cis*-cleavage. (a) SPR sensorgram of the interaction between Cas12a and the HPV-16-targeting sgRNA. The data were fit to a global 1:1 kinetic model (dashed lines) to determine *k*_on_ and *k*_off_. (b) *cis*-Cleavage of a dsDNA fluorescence reporter by Cas12a. The data were fit to a first-order kinetic equation to determine *k_cis_*. (c) Contour plots showing the concentration of cleaved reporter as a function of the rate of amplification (*k*_amp_) *vs* rate of *cis*-cleavage (*k*_*cis*_) for the RPA–Cas12a assay employing precomplexed RNP (i) and the RPA–Cas12a assay employing *in situ* complexation with *k*_form_ = (ii) 175,000 M^–1^·s^–1^, (iii) 17,500 M^–1^·s^–11^, and (iv) 1750 M^–1^·s^–1^. Target concentration was held at 0.2 copies/μL (c-i–iv). As the rate of RNP formation (*k*_form_) increases the parameter space in which the signal is detected decreases, as indicated by the area under the curve. (d) Contour plots showing the concentration of cleaved reporter as a function of the rate of amplification (*k*_amp_) *vs* the rate of *cis*-cleavage (*k*_*cis*_) for the RPA–Cas12a assay employing (i) precomplexed RNP and (ii) *in situ* complexation at a target concentration of 2 copies/μL. The rate of RNP formation (*k*_form_) was held at 175,000 M^–1^·s^–1^. When compared to the data at 0.2 copies/μL, the area under the curve is increased. For each dataset, time (*t*) was set at 60 min.

We next investigated the mechanisms behind the improved limit of detection observed for the *in situ* one-pot RPA–Cas12a assay. Specifically, we wanted to test the hypothesis that the inferior limits of detection observed with the traditional one-pot assays were attributable to the competition between the CRISPR-Cas12 *cis-*cleavage system and nucleic acid amplification, particularly during the early stages of the assay. Key to this was determining the rate-limiting step of *cis*-cleavage. We reasoned that double-stranded breaks would only occur when the sgRNA is correctly bound to the Cas12, that is, when the RNP is formed. Accordingly, we studied the kinetics of RNP formation using surface plasmon resonance (Figure 3a). Biotin-conjugated HPV16 sgRNA was attached to a streptavidin-modified carboxymethyl dextran-coated gold chip, and dilutions of Cas12a were analyzed under uniform flow. Response curves were globally fit to a 1:1 kinetic binding model to compute the association (*k*_on_ = 1.75 × 10^5^ M^–1^**·**s^–1^) and dissociation (*k*_off_ = 1.87 × 10^–4^ s^–1^) rate constants, as well as the dissociation equilibrium constant (*K*_D_ = 1.75 nM, defined as *k*_off_/*k*_on_). We analyzed interaction at different flow rates (10, 30, and 100 μL/min) and observed no significant changes in the association/dissociation rate constants, thus ruling out mass transport effects.

These data suggest that under the studied experimental conditions ([Cas12a] = 25 nM and [sgRNA] = 25 nM), it would take approximately 1050 s (17.5 min) to generate >95% of the maximum theoretical RNP concentration. However, several features of the SPR experiments are likely to impact the accuracy of these estimates. First, experiments were performed in a buffer that approximates the RPA–Cas12a buffer but does not perfectly replicate it. While we were able to ensure that pH and salt concentrations were identical, including all enzymes, accessory proteins and dNTPs were prohibitively expensive due to the large volumes of buffer required in SPR experiments. PEG was also omitted as this is incompatible with the SPR instrument. Binding kinetics are sensitive to differences in buffer composition, and interactions between the sgRNA and enzymes/accessory proteins are likely, given their opposing charges. Considering these factors, the calculated *k*_on_ value is likely to be higher than in our actual *in situ* RPA–Cas12a assay.

The omission of PEG is particularly problematic, as PEG causes a non-negligible viscosity change within the assay buffer, potentially impacting reagent diffusion. With this in mind, we studied the diffusion of Cas12a and the HPV-16 sgRNA using fluorescence correlation spectroscopy (FCS) in the exact *in situ* RPA-Cas12a assay buffer. We obtained diffusion coefficients of 16.7 ± 2.14 and 47.02 ± 2.7 μm^2^·s^–1^ for Cas12 and sgRNA, respectively. These values suggest that if RNP formation were diffusion-controlled, >95% formation would be achieved within 125 s within this buffer (Figure S2).^21^ This is significantly faster than the association kinetics measured *via* SPR (1050 s to reach >95% RNP formed), indicating that interaction is not diffusion-controlled under the assay conditions.

The next step in the *cis*-cleavage cascade is the binding of the RNP to the target and the subsequent introduction of a double-stranded break. To estimate the rate at which this occurs, target cleavage was studied under single-turnover conditions, with the concentration of the RNP in large excess (2 μM) over the target (20–80 nM) (Figure 3b). For these experiments, we employed a fluorescently tagged synthetic HPV-16 target with a black hole quencher strategically placed on the complementary strand, ten bases downstream of the Cas12a double-stranded break site. After successful cleavage, the quencher dissociates from the target, allowing *k*_obs_ to be calculated from the measured fluorescence emission. At 37 °C, we determined a *k*_obs_ of 0.0024 s^–1^. This value was essentially invariant to changes in the target concentration, suggesting that the rate-limiting step is not binding of the target by the RNP but rather a postbinding process, such as PAM recognition or target cleavage. This agrees with the mechanisms previously described in the literature.^16,22^

Next, we used these experimental values to construct mathematical models for both the *in situ* and precomplexed RPA–Cas12a assays (Scheme 1). We performed a multiparametric sweep, computing the concentration of the cleaved reporter (fluorescence) as a function of the rate of RNP formation (*k*_on_, henceforth denoted *k*_form_), the rate of *cis*-cleavage (*k*_*cis*_), and the rate of DNA amplification (*k*_amp_). For *k*_form_, we set an upper bound as the value obtained from SPR analysis (1.75 × 10^5^ M^–1^**·**s^–1^) (Figure 3c-ii). Since we reasoned that this value was likely higher than in our assay, we also modeled *k*_form_ values of 1.75 × 10^4^ and 1.75 × 10^3^ M^–1^**·**s^–1^ (Figure 3c-iii,iv). For *k*_*cis*_, the lower bound was set to the experimental value (0.0024 s^–1^) and the higher bound was set to twice the experimental value reported by Nalefski et al. (0.023 s^–1^).^16^ Due to the incompatibility of the RPA TwistAmp kit with target-specific fluorescent probes, it was impossible to determine the rate of DNA amplification (*k*_amp_) experimentally. Further, reliable *k*_amp_ values were not found in the literature. Accordingly, we intentionally spanned a wide range of *k*_amp_ values between 100 and 18,000 M^–1^**·**s^–1^. Importantly, *k*_cis_ was divided by the initial concentration of Cas12a (25 nM) and converted into a bimolecular rate constant to respect the law of mass action. For the precomplexed assay, we assumed that the RNP was fully formed and thus divided it by the initial concentration of RNP (25 nM) instead. For the *in situ* assay, we used *k*_form_ to scale the concentration of RNP with time. For *trans*-cleavage of the reporter, we employed a cleavage rate (*k*_*trans*_) of 1.95 s^–1^, the value obtained previously for this system.^15^ We utilized the first-order rate constant, *k*_*trans*_, since the concentration of the reporter was held in large excess (2000 nM) and well above the *K*_m_ value (271 nM).^15^ Using the calibration curve presented in Figure S3, we set the threshold concentration for a detectable cleaved reporter at 668 pM. All cleaved reporter concentrations were calculated for an assay duration of 60 min. Initially, we applied the model to two target concentrations: 0.2 copies/μL (Figure 3c) and 2 copies/μL (Figure 3d). These concentrations were chosen since they represent the boundary at which the performance of the *in situ* and precomplexed assays begin to merge (as seen in Figure 2).

The model highlights several interesting features of both the precomplexed and *in situ* systems. At low target DNA concentrations, the model suggests that the concentration of the cleaved reporter is directly proportional to the rate of DNA amplification (*k*_amp_) but inversely proportional to the rate of *cis*-cleavage (*k*_*cis*_). At a target concentration of 0.2 copies/μL, both systems “theoretically” fail to produce detectable reporter concentrations when *k*_*cis*_ is high and *k*_amp_ is low. However, differences between the two systems become apparent when *k*_*cis*_ is low and *k*_amp_ is high. The model indicates that the parameter space in which a positive signal can be obtained is significantly greater for the *in situ* assay than for the precomplexed assay (Figure 3c-i,ii). Interestingly, the difference between the two assay formats grows as *k*_form_ decreases (Figure 3c-ii–iv). This is to be expected since a reduction in *k*_form_ results in a lower RNP concentration (especially at early reaction times). This means that even if each individual RNP cleaves DNA faster (higher *k*_*cis*_ values), the absolute amount of target cleavage is small enough to be outcompeted by DNA amplification. Increasing the target concentration to 2 copies/μL increases the theoretical parameter space in which a positive signal is observed for both *in situ* and precomplexed assays (Figure 3d).

To summarize, the developed model supports our hypothesis that at low target concentrations *cis*-cleavage can outcompete DNA amplification, resulting in the relatively poor detection limits observed in traditional one-pot RPA–Cas12a assays.^11,23^ The model further suggests that for any given target concentration and DNA amplification rate, a threshold *cis*-cleavage rate exists under which sufficient reporter is cleaved to generate a detectable signal. Since a lower RNP concentration is present at the start of the *in situ* assay, the rate of target cleavage remains under this threshold and a signal is observed. Conversely, when a precomplexed RNP is employed, the rate of target cleavage is above the threshold and no signal is observed. As the target concentration increases, so does this threshold—this explains why we observe a convergence in performance between the *in situ* and precomplexed assays as the target concentration increases, as seen in Figure 2.

## Conclusions

Practical and simple methods for minimizing the competition between *cis*-cleavage and nucleic acid amplification in NAAT–CRISPR-Cas assays are lacking, but critical, for increasing the adoption of NAAT–CRISPR-based assays. Our system achieves this in the simplest possible way by leveraging the relatively slow kinetics of RNP formation to limit the *cis* exonuclease activity of Cas12. By modeling this process, our work provides insight into the mechanisms underpinning RPA–CRISPR-Cas assays and highlights the benefits of purposefully limiting the *cis* exonuclease activity of Cas proteins. The competition between *cis* exonuclease target cleavage and polymerase-mediated target amplification is fundamental to all NAAT–CRISPR-Cas assays. Accordingly, although we studied an RPA–CRISPR-Cas12a system, we are confident that the results can be immediately extended and applied to other NAAT–CRISPR-Cas systems. The exception to this are systems in which the optimal temperatures for nucleic acid amplification and CRISPR-Cas detection differ substantially, for example, LAMP–CRISPR-Cas. However, given the advances made in engineering thermophilic Cas proteins to operate at temperatures above 60 °C (as is required by LAMP),^24^ it seems likely that our findings will soon be applicable to such systems.

Our findings have broad implications. Until now, it was commonly thought that achieving consistently low limits of detection in NAAT–CRISPR-Cas assays was only possible using two-step protocols. Our data indicate that the robust detection of low-titer targets is possible in true one-pot, one-step protocols, provided *cis* exonuclease activity can be minimized during early assay times. *Cis* exonuclease rates are dependent on both the kinetics of RNP formation and the subsequent introduction of double-stranded breaks in the target (*cis*-cleavage); this presents multiple areas for intervention and improvement. The association kinetics between sgRNAs and their Cas proteins could be deliberately perturbed by employing chemically modified sgRNAs or engineered Cas proteins or by using high ionic strength assay buffers. Engineering Cas proteins with slower *cis*-cleavage rates is another promising avenue of exploration.^5^ Perhaps most importantly, one-pot CRISPR-Cas assays are useful for the development of point-of-care CRISPR-Cas-based diagnostics, as they greatly simplify the testing process by removing pipetting and mixing steps. Given the persistent challenges of robustly integrating multistep protocols at the point of care, it seems likely that one-pot protocols will come to dominate this space. It would be very interesting to explore how this method could be integrated with existing technologies to create simpler, more accessible NAAT–CRISPR-Cas diagnostics (*i.e.*, sample in–result out point-of-care devices). We hope that the data and insights presented in this paper encourage others to explore these areas.

## Supporting Information Available

Details of the oligonucleotides used in this study; descriptions of the experimental protocols; and additional figures related to the computational modeling of the reaction (PDF)

## Notes

**Ethical Statement** This study was approved by the ethical committee of the University Hospital of Ghent (Belgium) under the reference 2019/1687. All participants provided written informed consent upon the start of the study for their samples to be used.
