# Supplementary material for: In Situ Complexation of sgRNA and Cas12a Improves the Performance of a One-Pot RPA–CRISPR-Cas12 Assay
Source: Anal Chem. 2024 Jun 12;96(25):10443–50. doi: 10.1021/acs.analchem.4c01777 (PMC11210716; doi:10.1021/acs.analchem.4c01777)
Supplement: Supplementary file 1 — ac4c01777_si_001.pdf [file ac4c01777_si_001.pdf]

Supporting Information

In situ complexation of sgRNA and Cas12a improves the performance of a one-pot RPA–CRISPR-Cas12 assay

Jake M. Lesinski[1], Thomas Moragues[1], Prerit Mathur[1], Yang Shen[2], Carolina Paganini[1], Léonard Bezinge[1], Bo Verberckmoes[3], Bodine Van Eenooghe[3], Stavros Stavrakis[1], Andrew J. deMello[1*] & Daniel A. Richards[1*]

[1]Institute for Chemical and Bioengineering, ETH Zurich, Vladimir-Prelog-Weg 1, 8093, Zürich, Switzerland. [2]Institute of Food, Nutrition and Health, ETH Zurich, Schmelzbergstrasse 7, 8092, Zürich, Switzerland. [3]Faculty of Medicine and Health Sciences, Department of Public Health and Primary Care, Ghent University, De Pintelaan 185, 9000 Gent, Belgium.

* daniel.richards@chem.ethz.ch, andrew.demello@chem.ethz.ch,

Contents

TECHNOLOGY COMPARISON **3**

METHODS **4**

OLIGONUCLEOTIDES 4

ADDITIONAL PROTOCOLS 5

Preparation of HOLMES Buffer *5*

Preparation of SPR running buffer *5*

Preparation of RPA reaction buffer *5*

Expression of LbCas12a Enzyme *5*

Generation of target DNA *5*

Conjugation of Cas12 with Alexa Fluor 488 *6*

Plotting of Figure 2 *6*

Time-to-result determination *6*

Reaction modelling *6*

Diffusion model – sgRNA and Cas12a *7*

Establishing a detection threshold *8*

DECLARATION OF DATA LOCATION 9

REFERENCES **9**

Technology Comparison

Table S1. A collection of NAAT–CRISPR-Cas one-pot diagnostics assays that increase sensitivity by mitigating the competition between amplification and detection.

Assay	Mechanism to mitigate competition between CRISPR and NAAT systems	Analytical Performance	Comments	Reference
**Photocontrolled CRISPRCas12a System**	Photocaging of sgRNA prevents RNP formation	1 copy/µL (DNA, 90 min)	Results cannot be observed before photorelease	1
**Photoactivatable CRISPR/Cas12a-assisted RPA assay**	Photocaging of sgRNA prevents RNP formation	2.5 copies/µL (DNA, 40 min)	Results cannot be observed before photorelease	2
**Light-start CRISPRCas12system**	Photocaging of sgRNA prevents RNP formation	0.1 copies/µL (RNA, 30-40 min); 0.1 copy/µL (DNA, 10-20 min)	Results cannot be observed before photorelease	3
**Glycerol enhanced one-pot RPA CRISPR/Cas12a method**	Phase separation with viscous glycerol limits diffusion	10 copies/µL (RNA, 60 min)	Manual preparation/difficult implementation	4
**ORCas12a-BRV Assay**	Non-canonical/intentionally underperforming PAM limits *cis*-cleavage	100 copies/µL (DNA, 45 min)	Limited to finding and using non-canonical PAM that are slow but still specific	5
**sPAMC**	Non-canonical/intentionally underperforming PAM limits *cis*-cleavage	12 copies/µL (DNA, 15 min); 24 copies/µL (RNA, 25 min)	Limited to finding and using non-canonical PAM that are slow but still specific	6
**OAR-CRISPR Assay**	Asymmetric RPA process with sgRNA that targets ssDNA, eliminating competition for dsDNA	60 copies/µL (DNA, 8 min); 30 copies/µL (DNA, 12 min)	PAM-free, high limit of detection	7
**OCTOPUS**	Pipette separately and mix after initial amplification	1 CFU/mL in less than 50 min	Manual preparation/difficult implementation	8
**OR-DETECTR**	Pipette separately and mix after initial amplification	2.5 copies/µl input (30 minutes)	Manual preparation/difficult implementation	9
**In-situ complexed RPA/Cas12a One-pot**	By eliminating pre-complexation step to prevent immediate competition between Cas12a cis-cleavage and RPA amplification	0.2 copies/µl input (less than 40 minutes)	Increased simplicity with an order of magnitude decrease in limit-of-detection	This work

Methods

Oligonucleotides

Table S2. Sequences of the oligonucleotides used in this study. All oligonucleotides were commercially produced by Microsynth AG, Switzerland.

**Oligo Name**	**Sequence (5' to 3')**
**Fluorescence Reporter (trans-cleavage)**	FAM - TTATTATT - BHQ1
**Cis-cleavage reporter - quench**	GGCTTATATGTAGTTTCTGAAGTAGATATGGCAGCACATAATGACATCGG - BHQ1
**Cis-cleavage reporter - fluorophore**	FAM - CCGATGTCATTATGTGCTGCCATATCTACTTCAGAAACTACATATAAGCC
**HPV16 target DNA segment**	ATTATTTTCCTACACCTAGTGGTTCTATGGTTACCTCTGATGCCCAAATATTCAATAAACCTTATTGGTTACAACGAGCACAGGGCCACAATAATGGCATTTGTTGGGGTAACCAACTATTTGTTACTGTTGTTGATACTACACGCAGTACAAATATGTCATTATGTGCTGCCATATCTACTTCAGAAACTACATATAAAAATACTAACTTTAAGGAGTACCTACGACATGGGGAGGAATATGATTTACAGTTTATTTTTCAACTGTGCAAAATAACCTTAACTGCAGACGTTATGACATACATACATTCTATGAATTCCACTATTT
**HPV16 crRNA guide 1[10]**	UAAUUUCUACUAAGUGUAGAUUGAAGUAGAUAUGGCAGCAC
**HPV16 sgRNA guide – FAM conjugated**	FAM - UAAUUUCUACUAAGUGUAGAUUGAAGUAGAUAUGGCAGCAC
**biotin conjugated - HPV16 sgRNA guide**	UAAUUUCUACUAAGUGUAGAUUGAAGUAGAUAUGGCAGCAC - Biotin
**HPV16 RPA Primer Forward [10]**	TTGTTGGGGTAACCAACTATTTGTTACTGTT
**HPV16 RPA Primer Reverse [10]**	CCTCCCCATGTCGTAGGTACTCCTTAAAG
**Cleaved Fluorescence Reporter**	FAM - TTATTATT

Additional Protocols

Preparation of HOLMES Buffer

A 10x HOLMES buffer was prepared by combining the following reagents: 20 mM Spermidine, 400 mM Tris-HCl, 60 mM $MgCl_2$, 10mM DTT, 400 mM Glycine, 0.01% Triton X-100 (w/v), and 4% PEG-20,000 (w/v)[11,12]. The pH was then adjusted to 8.5 with potassium acetate.

Preparation of SPR running buffer

Due to the large volumes necessary for SPR experiments, an SPR running buffer was created by adding the following reagents to a final concentration of 25mM Tris, 100mM Potassium acetate, 2mM Dithiothreitol, and 20mM Magnesium acetate.[13] Nuclease-free water (Thermo Fischer Scientific, Waltham, USA) was added and the pH adjusted to 7.9 using potassium hydroxide.

Preparation of RPA reaction buffer

The RPA reaction buffer was the SPR running buffer with the addition of 5% PEG to mimic the RPA reaction mixture.[13]

Expression of LbCas12a Enzyme

The LbCas12a enzyme was expressed in *E. coli* BL21-GOLD (DE3) cells using an expression vector containing the DNA sequence for LbCas12a with an N-terminal 6xHis-tag and a C-terminal cysteine residue (Twist Bioscience, USA). Cells were cultured in LB media at 37°C until an OD of 0.5 was reached. Then, protein expression was induced with 0.5 mM isopropyl D-thiogalactopyranoside (99%, PanReac – AppliChem, Darmstadt, Germany), and the culture allowed to grow for 20 hours at 20°C. Cells were harvested, resuspended in lysis buffer (50 mM Tris-HCl, 500 mM NaCl, 5% (v/v) glycerol, 1 mM TCEP, 0.5 mM PMSF, 10 mM imidazole, pH 7.5) and lysed by sonication. The recombinant protein in the soluble fraction of the lysate was isolated using immobilized metal ion affinity chromatography (Cytiva, Marlborough, USA) and then further purified by size exclusion chromatography (HiLoad 16/600 Superdex 200 pg - Cytiva, Marlborough, USA) using a running buffer consisting of 20 mM Tris-HCl, 250 mM NaCl, 1mM TCEP, 5% (v/v) glycerol at pH 7.5. Finally, protein was transferred into a storage buffer prepared in nuclease-free conditions (50 mM Tris-HCl, 500 mM NaCl, 5% (v/v) glycerol, 1 mM TCEP, pH 7.5) using an Amicon-15 centrifugal filter (50 kDa MWCO - RC membrane, Merck Millipore, Burlington, USA), concentrated, aliquoted and stored at -80°C.

Generation of target DNA

Target DNA was produced by combining the following reagents (as final concentrations) as follows: ThermoScientific DreamTaq Hot Start PCR Master Mix (2X) 1x (Thermo Fischer Scientific, Waltham, USA), HPV16 RPA Primer Forward 500 nM, HPV16 RPA Primer Reverse 500 nM, template DNA 250 aM and nuclease-free water. The thermal routine utilized was as follows: initial denaturation of 95°C for 2 minutes, then 40 cycles of denaturation (95°C for 30 seconds), annealing (62.3°C for 30 seconds), and elongation (72°C for 1 minute). A final elongation step of 72°C for 1 minute was performed.

PCR Evaluation of Clinical Samples

Clinical samples were collected by a gynecologist using a Viba brush (Rovers Medical Devices, Oss, Netherlands). The cervix and the superficial vaginal canal were swabbed with the brush, which then was rinsed

in Hologic ThinPrep medium (Hologic, Mississauga, Canada). DNA was extracted from the ThinPrep medium using a STARMAG 96 x 4 Universal Cartridge Kit (Seegene, Seoul, Republic of Korea), and analyzed using the Allplex HPV28 and Anyplex HPV HR detection assays on a Microlab STAR (Hamilton, Reno, USA) device equipped with a thermal cycler.

Table S3. Analysis of patient-derived samples using Allplex and Anyplex.

Sample	Allplex	Anyplex	Allplex Final Result (*Ct*)
**1**	Positive for HPV16	16(+++)	16 (21,63)
**2**	Positive for HPV16	16(++)	16 (32,70)
**3**	Positive for HPV16	16(++)	16 (29,38)
**4**	Positive for HPV16	16(++)	16 (33,66)
**5**	Positive for HPV16	16(++)	16 (30,80)
**6**	Positive for HPV16	16(++)	16 (28,35)
**7**	Positive for HPV16	16(+++)	16 (21,26)
**8**	Positive for HPV16	16(++)	16 (34,39)

Conjugation of Cas12 with Alexa Fluor 488

The protein was fluorescently labelled using a Lightning-Link Alexa Fluor 488 Fast Conjugation Kit (Abcam, Cambridge, U.K.), following the manufacturer's instructions. In brief, LbCas12a was diluted with a high-salt buffer (20 mM sodium phosphate, 500 mM NaCl, pH 7.5) to yield a final concentration of 1 mg mL^{-1}. Then, 100 µL of the protein dilution was added to the conjugation kit and the mixture incubated for 15 min at room temperature. The reaction was stopped by the addition of a quencher.

Plotting of Figure 2

Plots (a) and (b) in Figure 2 show a mean line (linearly interpolated between data points) with the shaded error range indicating ±3 standard deviations. Although the reading was not continuous (measurement every 2 minutes) the data is represented in this way for clarity. Plot (c) in Figure 2 shows all data points found (3 per concentration per assay format). The TTR was found as described below in the section "Time-to-result determination".

Time-to-result determination

The time-to-result algorithm was based on the work of Pena *et al,*.[14] which states that the "*The maximal slope value for the negative samples in an experiment was determined, and the SD of the slopes for the negative samples was calculated. The maximal slope value plus three SD values calculated from the negative samples was set as the cutoff value. The TTR (in minutes) was defined as the time at which the fluorescence of a sample surpassed the cutoff value in three consecutive recordings.*"

Reaction modelling

To deduce reasonable values for k_{amp}, we modelled the amplification rates alone, without the influence of Cas-mediated processes. These rates were found to span a region that illustrates amplification times in accordance with those found in the literature and the TwistDx manual.[15–17] These times were characterized by when the amplification reaction is able to move from its initiation phase to its exponential phase (**Figure S1**).

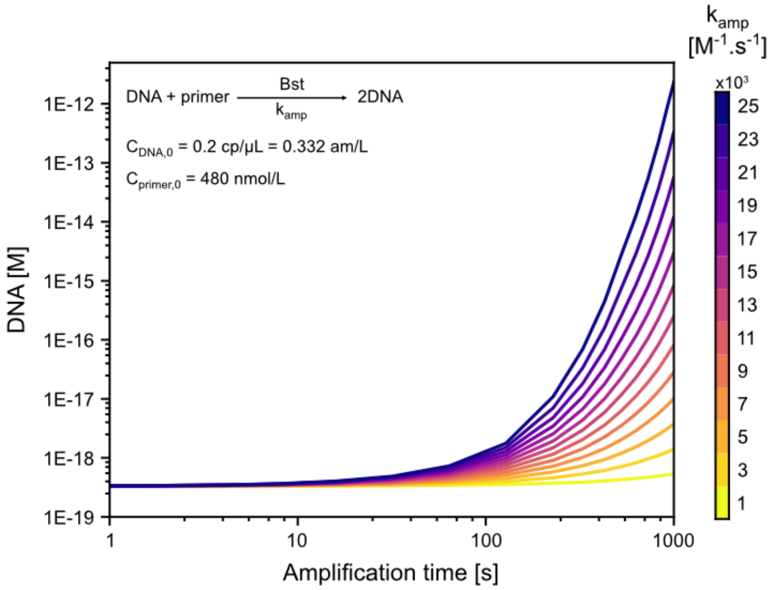

Figure S1. Modelling amplification times alone (without the addition of Cas proteins) to find reasonable doubling times.

Diffusion model – sgRNA and Cas12a

Utilizing a version of the Smoluchowski derivation of the Arrhenius equation (**Equation S1**),[18] we solved for the percentage of Cas12 and sgRNA experiencing a collision with each other across critical early-reaction times (**Figure S2**).

$$Z = (1000N_A)^{4/3} 8\pi^{-1} A\beta D_r ([A] + [B])^{1/3} [A][B] \tag{1}$$

Terms specific to **Equation S1** include A, β, D_r, [A] and [B] which represent the cross-sectional area of molecule A, the unitless fraction of the area of molecule B, the summed diffusion coefficient of molecules A and B, the concentration of molecule A in the reaction, and the concentration of molecule B in the reaction, respectively. Molecule A was chosen to be Cas12a. The radius of Cas12a used (to calculate the area "A") was taken as 3.7 nm as described by Bonini.[19] The fraction of molecule B (sgRNA) that is able to bind to molecule A was assumed to be 0.51. This fraction represents the portion of the sgRNA that forms the repeat or scaffold, the portion of the sgRNA that is involved in binding to the Cas12a.[10] "D_r" was the summation of diffusion coefficients from FCS, found to be 16.7 ± 2.14 μm^2/s and 47.02 ± 2.7 μm^2/s, for the Cas12a and sgRNA, respectively. The shaded region in plot a) indicates the error found in FCS (diffusion coefficient error for Cas12a = ± 2.14 μm^2/s and diffusion coefficient error for the sgRNA = ± 2.7 μm^2/s) propagated through the "D_r" term in accordance with the standard propagation of error equation, below.

$$\sigma = \sqrt{\sigma_A^2 + \sigma_B^2 - 2ab\sigma_{AB}}$$

σ_A, σ_B, and $\sigma_{A,B}$ are defined as the standard deviation of error of the diffusion coefficient of molecule A, molecule B, and the covariance of molecules A & B, respectively. The covariance term was assumed to be zero for the effect of one particle's diffusion coefficient on the other when unbound.

A key assumption in this discrete solution is that in each instance of a Cas12a and sgRNA collision, they are assumed to have bound, focusing the investigation on diffusion rather than orientation and collision energy. Data

indicate minimal delay before the reaction reaches its final concentration of RNP; this suggests that complex formation is not diffusion-controlled.

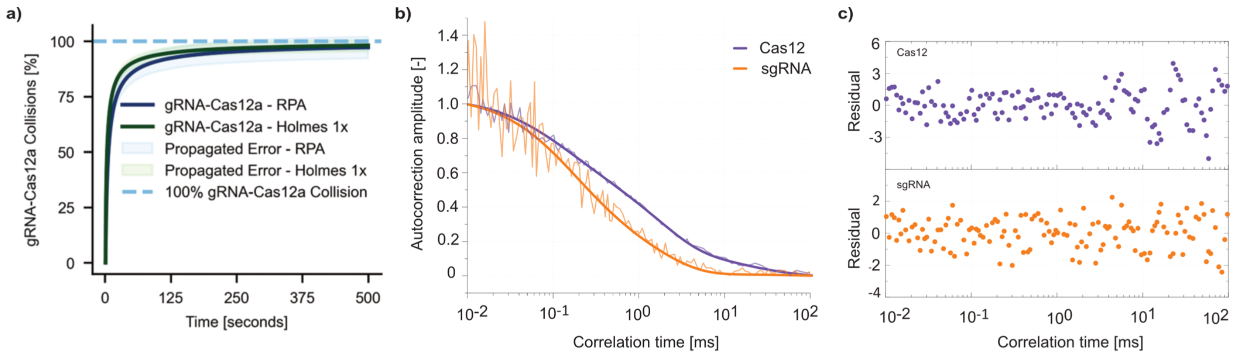

Figure S2. a) Modelling diffusion times according to the Smoluchowski derivation of the Arrhenius equation, as described above. b) FCS curve fits to determine diffusion coefficients. Normalized autocorrelation curves for both the sgRNA and Cas12a are overlaid with theoretical FCS curve fit. c) Residuals obtained from the curve fit for both the components, demonstrating the suitability of the model.

The code from this analysis is available at: https://github.com/hbdadboy/In-situ-Complexation-Improves-One-Pot.

Establishing a detection threshold

To understand what constitutes a detectable concentration of target in our model, a detection threshold was established on the plate reader where the data for **Figures 2 and 3** were collected. A reaction was created with the exact reagents and final concentrations as the precomplexed reaction above, with the following modifications: omission of target DNA and inclusion of cleaved fluorescent reporter (**SI Table 1**). The standard (uncleaved) fluorescence reporter was included in the solution as a control. The cleaved fluorescent reporter was titrated into reaction mixtures at final concentrations of 100 nM, 80 nM, 60 nM, 40 nM, 20 nM and 0 nM. From these concentrations, a line of best fit was constructed. The detection threshold was determined as the intersection of this line with the 99.7% confidence interval set around the mean of the zero value (0 nM of cleaved reporter).

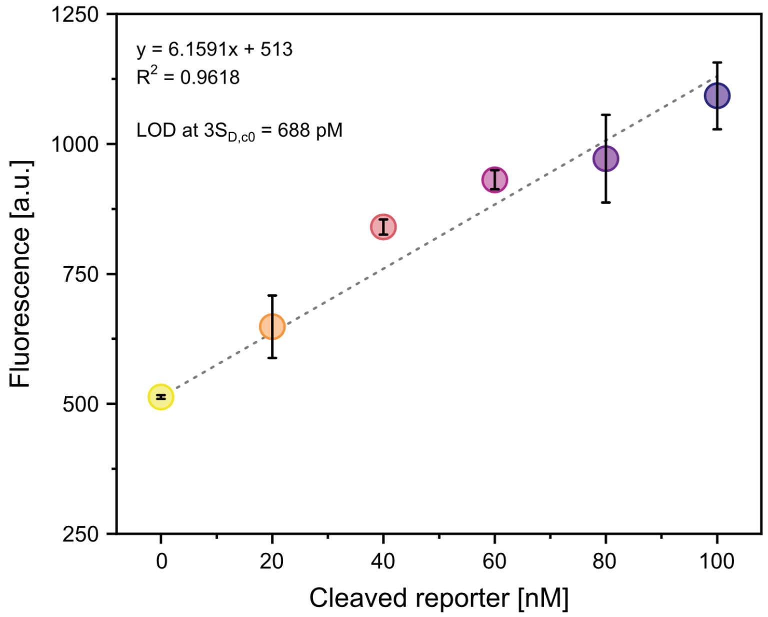

Figure S3. A calibration curve to establish the detection threshold for the cleaved reporter.

Declaration of data location

Data generated from this paper is can be found on github (https://github.com/hbdadboy/In-situ-Complexation-Improves-One-Pot).

References

(1) Hu, M.; Qiu, Z.; Bi, Z.; Tian, T.; Jiang, Y.; Zhou, X. Photocontrolled CrRNA Activation Enables Robust CRISPR-Cas12a Diagnostics. *Proc. Natl. Acad. Sci.* **2022**, *119* (26), e2202034119.

(2) Li, Q.-N.; Wang, D.-X.; Chen, D.-Y.; Lyu, J.-A.; Wang, Y.-X.; Wu, S.-L.; Jiang, H.-X.; Kong, D.-M. Photoactivatable CRISPR/Cas12a Sensors for Biomarkers Imaging and Point-of-Care Diagnostics. *Anal. Chem.* **2024**, *96* (6), 2692–2701.

(3) Hu, M.; Liu, R.; Qiu, Z.; Cao, F.; Tian, T.; Lu, Y.; Jiang, Y.; Zhou, X. Light-Start CRISPR-Cas12a Reaction with Caged CrRNA Enables Rapid and Sensitive Nucleic Acid Detection. *Angew. Chem. Int. Ed.* **2023**, *62* (23), e202300663.

(4) Lin, M.; Yue, H.; Tian, T.; Xiong, E.; Zhu, D.; Jiang, Y.; Zhou, X. Glycerol Additive Boosts 100-Fold Sensitivity Enhancement for One-Pot RPA-CRISPR/Cas12a Assay. *Anal. Chem.* **2022**, *94* (23), 8277–8284.

(5) Wang, P.; Guo, B.; Zhang, X.; Wang, Y.; Yang, G.; Shen, H.; Gao, S.; Zhang, L. One-Pot Molecular Diagnosis of Acute Hepatopancreatic Necrosis Disease by Recombinase Polymerase Amplification and CRISPR/Cas12a with Specially Designed CrRNA. *J. Agric. Food Chem.* **2023**, *71* (16), 6490–6498.

(6) Lu, S.; Tong, X.; Han, Y.; Zhang, K.; Zhang, Y.; Chen, Q.; Duan, J.; Lei, X.; Huang, M.; Qiu, Y.; Zhang, D.-Y.; Zhou, X.; Zhang, Y.; Yin, H. Fast and Sensitive Detection of SARS-CoV-2 RNA Using Suboptimal Protospacer Adjacent Motifs for Cas12a. *Nat. Biomed. Eng.* **2022**, *6* (3), 286–297.

(7) Yang, L.; Chen, G.; Wu, J.; Wei, W.; Peng, C.; Ding, L.; Chen, X.; Xu, X.; Wang, X.; Xu, J. A PAM-Free One-Step Asymmetric RPA and CRISPR/Cas12b Combined Assay (OAR-CRISPR) for Rapid and Ultrasensitive DNA Detection. *Anal. Chem.* **2024**, *96* (14), 5471–5477.

(8) Wang, Y.; Ke, Y.; Liu, W.; Sun, Y.; Ding, X. A One-Pot Toolbox Based on Cas12a/CrRNA Enables Rapid Foodborne Pathogen Detection at Attomolar Level. *ACS Sens.* **2020**, *5* (5), 1427–1435.

(9) Sun, Y.; Yu, L.; Liu, C.; Ye, S.; Chen, W.; Li, D.; Huang, W. One-Tube SARS-CoV-2 Detection Platform Based on RT-RPA and CRISPR/Cas12a. *J. Transl. Med.* **2021**, *19* (1), 74.

(10) Chen, J. S.; Ma, E.; Harrington, L. B.; Da Costa, M.; Tian, X.; Palefsky, J. M.; Doudna, J. A. CRISPR-Cas12a Target Binding Unleashes Indiscriminate Single-Stranded DNase Activity. *Science* **2018**, *360* (6387), 436–439.

(11) Lv, H.; Wang, J.; Zhang, J.; Chen, Y.; Yin, L.; Jin, D.; Gu, D.; Zhao, H.; Xu, Y.; Wang, J. Definition of CRISPR Cas12a Trans-Cleavage Units to Facilitate CRISPR Diagnostics. *Front. Microbiol.* **2021**, *12*, 766464
(12) Li, S.-Y.; Cheng, Q.-X.; Wang, J.-M.; Li, X.-Y.; Zhang, Z.-L.; Gao, S.; Cao, R.-B.; Zhao, G.-P.; Wang, J. CRISPR-Cas12a-Assisted Nucleic Acid Detection. *Cell Discov.* **2018**, *4*, 20.
(13) Li, J.; Macdonald, J.; Stetten, F. von. Review: A Comprehensive Summary of a Decade Development of the Recombinase Polymerase Amplification. *Analyst* **2019**, *144* (1), 31–67.
(14) Pena, J. M.; Manning, B. J.; Li, X.; Fiore, E. S.; Carlson, L.; Shytle, K.; Nguyen, P. P.; Azmi, I.; Larsen, A.; Wilson, M. K.; Singh, S.; DeMeo, M. C.; Ramesh, P.; Boisvert, H.; Blake, W. J. Real-Time, Multiplexed SHERLOCK for in Vitro Diagnostics. *J. Mol. Diagn.* **2023**, *25* (7), 428–437.
(15) Fan, X.; Li, L.; Zhao, Y.; Liu, Y.; Liu, C.; Wang, Q.; Dong, Y.; Wang, S.; Chi, T.; Song, F.; Sun, C.; Wang, Y.; Ha, D.; Zhao, Y.; Bao, J.; Wu, X.; Wang, Z. Clinical Validation of Two Recombinase-Based Isothermal Amplification Assays (RPA/RAA) for the Rapid Detection of African Swine Fever Virus. *Front. Microbiol.* **2020**, *11*, 1696
(16) Daher, R. K.; Stewart, G.; Boissinot, M.; Bergeron, M. G. Recombinase Polymerase Amplification for Diagnostic Applications. *Clin. Chem.* **2016**, *62* (7), 947–958.
(17) Lutz, S.; Weber, P.; Focke, M.; Faltin, B.; Hoffmann, J.; Müller, C.; Mark, D.; Roth, G.; Munday, P.; Armes, N.; Piepenburg, O.; Zengerle, R.; Stetten, F. von. Microfluidic Lab-on-a-Foil for Nucleic Acid Analysis Based on Isothermal Recombinase Polymerase Amplification (RPA). *Lab. Chip* **2010**, *10* (7), 887–893.
(18) Chen, J. Why Should the Reaction Order of a Bimolecular Reaction Be 2.33 Instead of 2? *J. Phys. Chem. A* **2022**, *126* (51), 9719–9725.
(19) Bonini, A.; Poma, N.; Vivaldi, F.; Biagini, D.; Bottai, D.; Tavanti, A.; Di Francesco, F. A Label-Free Impedance Biosensing Assay Based on CRISPR/Cas12a Collateral Activity for Bacterial DNA Detection. *J. Pharm. Biomed. Anal.* **2021**, *204*, 114268.
